# Exploring intensity-dependent modulations in EEG resting-state network efficiency induced by exercise

**DOI:** 10.1007/s00421-021-04712-6

**Published:** 2021-05-18

**Authors:** Daniel Büchel, Øyvind Sandbakk, Jochen Baumeister

**Affiliations:** 1 Exercise Science and Neuroscience Unit, Department of Exercise & Health, Faculty of Science, Paderborn University, Paderborn, Germany; 2 Department of Neuromedicine and Movement Science, Centre for Elite Sports Research, Norwegian University of Science and Technology, Trondheim, Norway

**Keywords:** Electroencephalography, Network efficiency, Exercise load, Resting-state network, Readiness, Functional connectivity

## Abstract

**Purpose:**

Exhaustive cardiovascular load can affect neural processing and is associated with decreases in sensorimotor performance. The purpose of this study was to explore intensity-dependent modulations in brain network efficiency in response to treadmill running assessed from resting-state electroencephalography (EEG) measures.

**Methods:**

Sixteen trained participants were tested for individual peak oxygen uptake (VO_2 peak_) and performed an incremental treadmill exercise at 50% (10 min), 70% (10 min) and 90% speed VO_2 peak_ (all-out) followed by cool-down running and active recovery. Before the experiment and after each stage, borg scale (BS), blood lactate concentration (B_La_), resting heartrate (HR_rest_) and 64-channel EEG resting state were assessed. To analyze network efficiency, graph theory was applied to derive small world index (SWI) from EEG data in theta, alpha-1 and alpha-2 frequency bands.

**Results:**

Analysis of variance for repeated measures revealed significant main effects for intensity on BS, B_La_, HR_rest_ and SWI. While BS, B_La_ and HR_rest_ indicated maxima after all-out, SWI showed a reduction in the theta network after all-out.

**Conclusion:**

Our explorative approach suggests intensity-dependent modulations of resting-state brain networks, since exhaustive exercise temporarily reduces brain network efficiency. Resting-state network assessment may prospectively play a role in training monitoring by displaying the readiness and efficiency of the central nervous system in different training situations.

## Introduction

Increasing load during endurance sports challenges both the cardiovascular and the central nervous system. While a well-adapted cardiovascular system is required to sufficiently supply the working muscles with metabolic energy during exercise, a highly developed interaction within the central nervous system is mandatory for motor coordination between upper-limb, trunk and lower-limb muscles (Bucher et al. 2018). In this regard, a precise sequential order of movements is crucial for the athlete to move efficiently during exercise, especially in technically demanding endurance sports (Holmberg et al. 2005). However, several investigations observed that high exercise load can impair the sequential coordination of limb movement, which consequently leads to reduced power output and reduced performance (Bassan et al. 2015; Bucher et al. 2018; Zory et al. 2009). It has been suggested that such effects can be explained by modulations in both central and peripheral neural circuits leading to impaired coupling of sensory perception and motor execution (McMorris et al. 2015). On the central site, stress-stimulated adreno-receptors seem to inherently modulate prefrontal cortex activity, which is a crucial instance for movement execution (Arnsten 2009). Moreover, metabolic products like blood lactate seem to modulate the excitability of cortical neurons (Magistretti and Allaman 2018). On the peripheral site, phenomena like reduced motoneuron excitability and reduced motor unit firing rates seem to dampen motor output (Taylor et al. 2016). Consequently, both reduced contractile function and modulated cortical processing may therefore impair motor coordination.

While the peripheral factors to loss of motor coordination after exhaustive exercise seem well understood, less is known on the central, cortical contributions. The few existing studies investigating cortical mechanisms related to exercise are majorly applying electroencephalography (EEG) as it is the most mobile and low-cost technique to quantify neural activity (Mehta and Parasuraman 2013). Under laboratory conditions, both up- and downregulations of cortical activity in sensorimotor brain areas in response to exhausting exercise were reported. For instance, modulations of activity in the frontal and sensory cortex after cardiovascular exhaustion in subjects performing a knee-angle reproduction task were observed (Baumeister et al. 2012). Further, EEG data recorded in response to incremental cycling exercise reveal bidirectional modulations of cortical activation in the frontal and sensory cortex (Brümmer et al. 2011; Robertson and Marino 2015). Interestingly, these modulations of regional EEG activity seem to even persist at rest after exercise, but do not reveal a clear intensity-dependent region-specific or frequency-specific pattern (Crabbe and Dishman 2004; Gramkow et al. 2020). Taking into account that the human brain is characterized by functional integration of multimodal information from regionally distinct brain areas by means of large-scale networks (Fox et al. 2005), the analysis of regional activity levels may not sufficiently reflect exercise-induced modulations of cortical mechanisms and could explain these heterogeneous findings.

Consequently, the assessment of brain resting-state networks (RSN) seems promising to detect cortical contributions to exhaustion-induced modulations of motor coordination. RSN are defined as regionally distinct brain structures which are functionally connected at rest (Shaw et al. 2015). The assessment of RSNs has evoked interest as they seem to reflect the responsiveness of the brain towards external stimuli (Raichle 2011). More precisely, it is suggested that modulations of RSN display ongoing and organized changes in excitability of neural ensembles. In the context of motor coordination and sports performance, RSN might therefore be a valuable tool to monitor the interconnectedness of attentional and sensorimotor brain areas as a measure of “readiness” for sports activity. Evidence for exercise-induced changes in RSN is revealed from functional magnetic resonance imaging (fMRI) studies reporting modulations in attention-related and sensorimotor brain networks (Rajab et al. 2014; Schmitt et al. 2019; Weng et al. 2016). Interestingly, RSN changes seem to better display intensity-dependent modulations of brain function than the regional assessment of brain activity due to EEG, as a down-regulation of the interconnectedness in sensorimotor brain networks was particularly reported after exhaustive exercise (Schmitt et al. 2019). Nevertheless, despite its advantages with regard to spatial resolution, fMRI remains unfavorable in exercise scientific settings due to the high costs, low temporal resolution, low mobility and long preparation time (Mehta and Parasuraman 2013). Therefore, moving towards RSN assessments using mobile EEG seems reasonable to get closer to the exercise load itself due to a faster recording in ecological exercise settings on the track at a low-cost level (Park et al. 2015).

RSN derived from EEG rely on the temporal coherence of electrical oscillations at different scalp locations and are expressed by means of functional connectivity (Imperatori et al. 2019). Based on functional connectivity measures, a brain graph can be constructed, where EEG sensors are treated as network nodes and the connections between two nodes are treated as edges (Farahani et al. 2019; Sporns 2013). Through that, brain graphs reveal information on how single nodes are interconnected and help to understand brain network organization in distinct mental, physiological or pathological conditions (Sporns 2013). Prominent outcomes describing network organization are the clustering coefficient (CC) displaying cortical segregation, the characteristic path length (PL) displaying global integration, and the small world index (SWI) describing brain network efficiency as a ratio of CC and PL (Kaminski and Blinowska 2018). An efficient brain network is expected to display a high SWI, characterized by a high CC and a low PL (Stam et al. 2014). To date, graph measures are majorly applied to display the function of the central nervous system in clinical investigations. In this regard, it is reported that healthy controls distinguish from Alzheimer’s patients (Vecchio et al. 2017), respectively, depression patients (Sun et al. 2019) by means of a higher SWI, displaying higher network efficiency. More precisely, a higher SWI at rest is regarded to express superior brain functions, as information in the central nervous system can be transferred more efficiently from any point of the network to another (Vecchio et al. 2017). Vice versa, reductions in SWI, referred to as network randomization, express a loss of network efficiency and may explain impaired brain functions in special populations (Peraza et al. 2018). Just recently, graph measures were applied in exercise-related settings during incremental cycling exercise. Findings demonstrate that network efficiency first increases intra-individually from low- to moderate-intensity exercise, while exhaustive exercise conditions seem to evoke loss of network efficiency (Porter et al. 2019; Tamburro et al. 2020). Moreover, a recent study reports exercise modality-specific changes of small-world characteristics after a dance training intervention in elderly citizens (Zilidou et al. 2018). Taken together, graph measures might be a valuable tool to display changes in network efficiency inter-individually and rather index the responsiveness of athletes to perform motor tasks. In this way, the EEG resting state provides a low-cost, fast-applicable opportunity to derive valuable information on athletes and patients under standardized conditions.

The aim of the present study was to explore the effect of exercise intensity induced by treadmill running on RSN efficiency derived from EEG-based graph analysis in a within-subject design. It was hypothesized that a loss of RSN efficiency could be observed after exhaustive exercise, indexed by a reduced SWI. For our explorative approach, we investigated the three frequency bands of theta, alpha-1 and alpha-2, as these have been previously shown to be modulated by exercise (Porter et al. 2019; Tamburro et al. 2020). Additionally, regional power spectral density was calculated to compare our results to previous publications demonstrating increased activity of brain regions after exercise (Crabbe and Dishman 2004).

## Methods

### Participants

Sixteen physically active male subjects participated at the present investigation. All participants performed exercise at least three times a week and were used to perform endurance running exercise. Before the individual assessment started, each participant ran through a medical assessment including health history questionnaire, followed by a 12-lead resting electrocardiogram screened by a medical doctor of the department. Written consent was obtained from each participant and all investigations were conducted in accordance with the local ethics committee of Paderborn University. An overview of the participants’ physical characteristics is given in Table 1.

**Table 1:** Mean characteristics of the study sample (*n* = 22) presented as mean ± SD

Variables	
Age (years)	24.56 ± 3.3
Body mass (kg)	75.95 ± 9.7
Size (cm)	182.13 ± 8.7
HF_peak_ (bpm)	187.63 ± 9.9
B_La peak_ (mmol/l)	7.43 ± 1.8
VO_2 peak_ (ml/min/kg)	51.63 ± 5.6
vVO_2 peak_ (km/h)	17.86 ± 1.6
50% vVO_2 peak_ (km/h)	8.96 ± 0.8
70% vVO_2 peak_ (km/h)	12.51 ± 1.2
90% vVO_2 peak_ (km/h)	16.08 ± 1.5

*HF*_*pea*k_ peak averaged 10-s heart frequency measured during ramp protocol; *B*_*La peak*_ blood lactate value measured after ramp protocol cessation; *VO*_*2 peak*_ peak oxygen uptake averaged over the highest 1-min consecutive measurement; *vVO*_*2 peak*_ minimum running speed during the highest 1-min consecutive VO_2_ measurement; *% vVO*_*2 peak*_ relative running speed according to maximum oxygen uptake

### Peak oxygen uptake

On the first day of examination, peak oxygen uptake (VO_2 peak_) was assessed with an incremental ramp test while running on a motorized treadmill (h/p/cosmos Pulsar 3p; Traunstein, Germany) accompanied by a mobile spirometry analysis (Metalyzer 3B, Cortex Biophysik, Leipzig, Germany). Participants started running at 7 km/h for 4 min with a 1% gradient set throughout. After the initial 4 min, running speed increased by 1 km/h each minute until participants reached voluntary exhaustion. To determine exercise load with regard to the individual fitness level, VO_2 peak_ was calculated based on breath-by-breath gas exchange. VO_2 peak_ was defined as the peak oxygen uptake averaged over the highest 1-min consecutive measurement throughout the ramp protocol. As an external load equivalent, the minimum running speed during the highest 1-min consecutive VO_2_ measurement was determined as vVO_2 peak_. In addition, heart rate (HR) was assessed using a 12-lead electrocardiography (custo cardio 100 BT, customed, Ottobrunn, Germany) and peak HR (HR_peak_) was defined as the maximum 5-s average value measured throughout. As a further objective outcome of maximal physiological load, blood lactate concentration (B_La_) in mmol/ l was assessed after cessation of the ramp protocol. Blood samples were analyzed using the BIOSEN C-Line lactate analyzer (EKF-diagnostic GmbH, Barleben, Germany).

### Incremental treadmill protocol

**Fig. 1:**
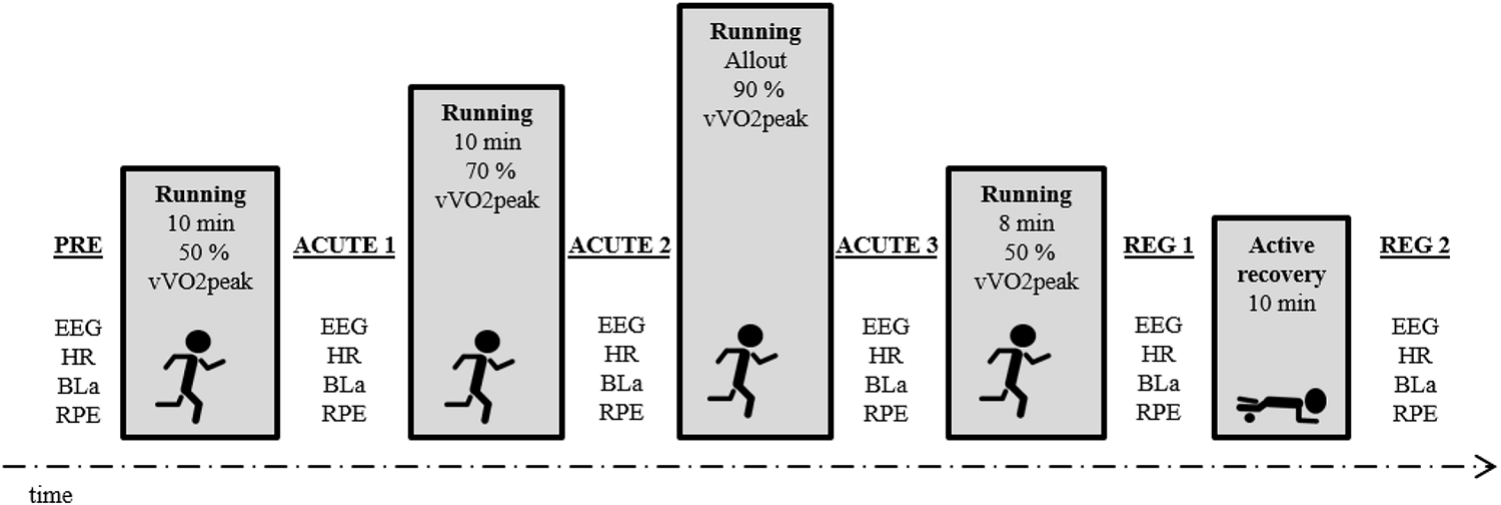
Experimental protocol. Participants performed 4 running stages at 50%, 70%, 90% and 50% of their individual speed at peak oxygen uptake assessed during the ramp protocol. At six different timepoints (PRE, ACUTE1, ACUTE2, ACUTE3, REG1 and REG2), electroencephalography (EEG) resting state in sitting position was recorded over 5 min. Further, resting heart rate over 5 min in sitting position (HR), blood lactate sample from the earlobe (B_La_) and rate of perceived exertion (RPE) were assessed

On the second examination day, subjects performed an incremental protocol while running on the treadmill. Based on individual vVO_2 peak_, three running intensities were determined; low (50% vVO_2 peak_), moderate (70% vVO_2 peak_) and exhaustive (90% vVO_2 peak_) intensity. The protocol started with running for 10 min at low intensity, followed by 10 min running at moderate intensity and rather followed by running until voluntary exhaustion. After the high-intensity stage, a stage of running at 50% vVO_2 peak_ for 8 min, followed by an active recovery phase of 10 min where participants were asked to stretch and/or foam roll. Physiological parameters were obtained and EEG resting states were recorded before low-intensity running (PRE), after low-intensity running (ACUTE1), after moderate-intensity running (ACUTE2), after exhaustive running (ACUTE3), after cool-down running (REG1) and after active recovery (REG2). An overview of the treadmill protocol is presented in Fig. 1.

### Physiological parameters

The physiological parameters obtained during the protocol were: B_La_ samples (20 μl) taken directly after each condition from the right earlobe; Borg Scale, asking the participant for subjective exhaustion, ranging from 6 (“very easy”) to 20 (“very hard”); resting average HR (HR_rest)_, obtained as 5-min average value during resting-state assessment and running HR (HR_run_) obtained as the overall average value during each incremental exercise stage, respectively, active recovery using an ECG sensor connected to a chest belt (Polar H10, Polar, Kempele, Finland).

### EEG assessment

Electrocortical activity was recorded by 65 passive wet electrodes (RNET, BrainProducts, Germany) connected to a wireless transmission system (LiveAmp, BrainProducts, Germany). Electrodes were online-referenced to FCz and attached according to the international 10–20 system. EEG were recorded from the participants in a sitting position for 5 min with eyes open in a sound attenuated room in front of a white wall. Due to the fast applicability of wet electrodes, resting-state EEG assessment started within 3.5 min after stage termination. Before starting the EEG recording, impedances were checked to be below 25 kΩ for all electrodes. As running on the treadmill at high intensities wearing an EEG cap is not comfortable, EEG cap was applied to the participant’s head within each break and was removed again after finishing the recording. To extract the corresponding ECG activity of the EEG resting-state time windows, timestamps were set manually using a watch (Polar M430, Polar Electronics, Kempele, Finland).

### EEG preprocessing

For processing, EEG data were imported into the EEGLAB toolbox v14.1.2 (Delorme and Makeig 2004) for MATLAB (Version R2019a, Mathworks Inc., Natick, USA). The EEG signals were first processed applying the CLEANLINE filter (Mullen 2011) at 60 and 120 Hz and band-pass-filtered applying a finite impulse response filter between 3 and 40 Hz. Then, the filtered data were re-referenced to common average and the reference-electrode FCz was recomputed. Further, the EEG signals were down-sampled from 500 to 256 Hz. To get rid of artifacts, signals were cleaned using the clean_rawdata EEGLAB plugin (Miyakoshi and Kothe 2014). By means of interpolating bad channels and applying automated subspace reconstruction (ASR), a component-based method was applied to effectively interpolate transient or large-amplitude artifacts. After cleaning the data, AMICA-independent component analysis algorithm (Palmer 2015) was applied to decompose the clean signal into brain signals and non-brain signals generated by muscle activity, eye activity, electrocardiogram and sweat and sources from non-brain signals were removed. The pruned data were epoched into sequences of 8 s with a phase overlap of 4 s. Previous studies stated that epochs shorter than 4 s should be avoided as they overestimate FC measures due to lack of variance in the data (Fraschini et al. 2016; Lai et al. 2018). The first 50 epochs of each EEG resting-state recording were chosen for power and connectivity analysis.

### EEG power analysis

To extract topographical activation on scalp level, fast Fourier transformation was applied on the epoched data to derive power spectral density at 10 Hz for the frequencies between 3 and 30 Hz. Theta (4–8 Hz), alpha-1 (8–10.5 Hz) and alpha-2 (10.5–13 Hz) were defined as frequency bands of interest. Power spectral density values were calculated in four midline ROIs assigned as frontal (FCz, FC1, FC2, Fz, F1, F2), central (Cz, C1, C2, CPz, CP1, CP2), parietal (Pz, P1, P2, POz, PO3, PO4) and occipital (Oz, O1, O2). Even though previous analyses revealed that changes in EEG power in response to exercise did not indicate systematic region-specific effects, the assignment to ROIs was done to provide a more specific description of brain activity in response to exercise compared to the global PSD values (Crabbe and Dishman 2004; Gramkow et al. 2020).

### EEG network analysis

To explore RSN organization, the epoched data were imported to the BrainWave software version 0.9.151.7.2 (Stam 2018). Functional connectivity between EEG signals was derived by means of the weighted phase-leg-index (wPLI), an extension to the phase leg index (PLI) which is reported to be less sensitive to noise (Vinck et al. 2011). The wPLI is an index of the asymmetry in the distribution of phase differences calculated from the instantaneous phases of two time series and ranges from 0 to 1, while 1 represents a persistent, asymmetric phase leg indicating functional interaction between two signals (Hardmeier et al. 2014). Thus, the wPLI deprives zero-phase lag relationships between two signals (Vinck et al. 2011). In this context, Sadaghiani and Wirsich (2019) suggested to choose connectivity measures depriving zero-phase data to avoid source leakage and false positive connectivity estimation due to spurious activity based on volume conduction for interpretation of sensor EEG data. Moreover, since phase-lag measures were reported to be insensitive from signal amplitudes (Stam et al. 2007), wPLI was used as it seems less sensitive to exercise-induced changes in EEG power and amplitude (Crabbe and Dishman 2004). The wPLI was computed on the scalp level for each possible connection between two electrodes for each epoch, resulting in 50 65-by-65 grids for each subject and each condition. The wPLI computation was performed on band-pass-filtered data in the previously defined frequency bands of interest theta, alpha-1 and alpha-2. The beta band was not investigated, as it is associated with task-related rather than resting-state modulations (Cheron et al. 2016) and is further sensitive to muscle-induced artefacts. Due to rejection of independent components associated with muscle activity, the manipulation of beta oscillations seems likely for the present dataset.

To derive graph-based brain network metrics, the wPLI matrices were imported to the MATLAB-based Brain Connectivity Toolbox (Rubinov and Sporns 2010). For the analysis of RSN, weighted undirected networks were chosen as they were suggested to give more specific information on the degree of connectivity between two nodes compared to binary networks (Telesford et al. 2017). Due to the weighted network approach, all possible connections within the network were kept and no binary connectivity threshold for edge reduction was set. For graph derivation, wPLI matrices were normalized, by converting all wPLI values to the range from 0 to 1 based on the real range of individual wPLI values to obtain inter-individual comparability. For each epoch and each condition, graph measures CC, PL and SWI were computed as global graph outcomes. Thus, CC and PL were derived as mean values of all individual channels across the scalp. CC and PL are provided as normalized values, where normalization was performed by dividing each individual value by the mean of all other values over all frequency bands (Vecchio et al. 2017). SWI is given as the individual ratio between normalized CC and normalized PL. An overview of the derived graph measures and definitions is presented in Table 2.

**Table 2:** Overview of calculated graph outcomes and definitions

Outcome	Interpretation	Interpretation	Reference
Clustering coefficient (CC)	Network segregation	Higher values indicate stronger tendency of a node to build clusters within its direct neighborhood	Rubinov and Sporns (2010)
Characteristic path length (PL)	Network integration	Higher values indicate stronger capacity of the network to become interconnected and exchange information	Rubinov and Sporns (2010)
Small world index (SWI)	Network efficiency	Higher values indicate more efficient network by communicating via many short and few long connections	Vecchio et al. (2017)

### Statistics

Statistical analyses were performed using SPSS 25 (SPSS Inc., Chicago, IL). All results are given as mean ± SD, normal distribution of the data was verified applying Kolmogorov–Smirnov test. Analysis of variances (ANOVA) for repeated measures was applied to compare physiological outcomes and resting-state EEG data between the different experimental conditions (PRE, ACUTE1, ACUTE2, ACUTE3, REG1 and REG2). Tests of sphericity (Mauchly) and homogeneity (Levenne) were applied to interpret the main effects. Post hoc tests corrected for multiple comparison according to Bonferroni–Holm were applied in case of significant main effects, to localize effects between the different experimental conditions. The level of significance was set at *p* < 0.05. For interpretation of effect sizes, partial eta square (partial eta^2^) was calculated and 0.01, 0.06, and 0.14 were considered as small, medium, and large effect sizes, respectively (Lakens 2013).

## Results

### Physiological assessment

The average time to cessation at the maximum voluntary exhaustion stage was 9.6 ± 3.0 min. Significant main effects for condition were observed for B_La_ (*p* < 0.001), RPE (*p* < 0.001), HR_rest_ (*p* < 0.001), and HR_run_ (*p* < 0.001). For all physiological outcomes assessed, maxima were recorded at ACUTE 3. An overview of the physiological responses to different exercise loads is given in Table 3.

**Table 3:** Mean (neuro-)physiological outcomes and corresponding standard deviations in response to different exercise intensities among 16 participants running on a treadmill, preceded by one 5-min baseline measurement at rest (PRE) and followed by five post-exercise measurements

	PRE	ACUTE 1	ACUTE 2	ACUTE 3	REG 1	REG 2	ANOVA
HR_run_ (in % of HR_peak_)	–	67.99 ± 5.0	80.14 ± 5.2	90.43 ± 3.6	76.33 ± 4.7	55.42 ± 6.6	*p* < 0.001; *F* = 733.8; part. eta^2^ = 0.98
HR_rest_ (in % of HR_peak_)	32.40 ± 6.3	38.78 ± 7.3	45.12 ± 6.7	53.92 ± 5.7	50.21 ± 5.5	44.84 ± 5.3	*p* < 0.001; *F* = 108.9; part. eta^2^ = 0.88
B_La_ (in mmol/l)	0.99 ± 0.4	1.36 ± 0.7	2.80 ± 1.3	7.85 ± 2.1	3.13 ± 1.3	1.67 ± 0.6	*p* < 0.001; *F* = 122.6; part. eta^2^ = 0.89
RPE	7.25 ± 1.6	11.13 ± 1.7	14.19 ± 2.1	18.88 ± 0.6	11.13 ± 2.0	8.19 ± 1.9	*p* < 0.001; *F* = 170.9; part. eta^2^ = 0.92
SWI theta	0.99 ± 0.1	1.02 ± 0.1	1.01 ± 0.1	0.96 ± 0.1	1.02 ± 0.1	1.01 ± 0.1	*p* = 0.022; *F* = 2.8; part. eta^2^ = 0.16
SWI alpha-1	0.96 ± 0.2	0.99 ± 0.2	1.03 ± 0.2	1.02 ± 0.2	1.09 ± 0.3	1.01 ± 0.2	*p* = 0.012; *F* = 3.2; part. eta^2^ = 0.17
SWI alpha-2	1.00 ± 0.1	1.01 ± 0.1	1.02 ± 0.1	1.03 ± 0.1	1.00 ± 0.1	0.98 ± 0.1	*p* = 0.397; *F* = 1.0; part. eta^2^ = 0.07
PL theta	1.01 ± 0.0	0.99 ± 0.0	1.00 ± 0.0	1.01 ± 0.0	0.99 ± 0.0	1.00 ± 0.0	*p* = 0.105; *F* = 1.9; part. eta^2^ = 0.11
PL alpha-1	1.02 ± 0.1	1.01 ± 0.1	0.99 ± 0.1	1.00 ± 0.1	0.98 ± 0.1	1.00 ± 0.1	*p* = 0.028; *F* = 2.7; part. eta^2^ = 0.15
PL alpha-2	1.00 ± 0.0	1.00 ± 0.0	0.99 ± 0.1	0.99 ± 0.0	1.01 ± 0.1	1.01 ± 0.0	*p* = 0.155; *F* = 1.7; part. eta^2^ = 0.10
CC theta	1.00 ± 0.0	1.01 ± 0.0	1.00 ± 0.1	0.97 ± 0.0	1.02 ± 0.0	1.00 ± 0.1	*p* = 0.008; *F* = 3.4; part. eta^2^ = 0.19
CC alpha-1	0.97 ± 0.1	0.98 ± 0.1	1.01 ± 0.1	1.00 ± 0.1	1.04 ± 0.2	1.00 ± 0.1	*p* = 0.011; *F* = 3.2; part. eta^2^ = 0.18
CC alpha-2	1.00 ± 0.1	1.00 ± 0.1	1.00 ± 0.1	1.01 ± 0.1	1.00 ± 0.1	0.99 ± 0.1	*p* = 0.690; *F* = 0.6; part. eta^2^ = 0.04

Measurements refer to 5-min resting-state recordings following 10-min low-intensity running (ACUTE 1), 10-min moderate-intensity running (ACUTE 2), all-out exhaustive running (ACUTE 3), 8-min cool-down running (REG 1) and 10-min active recovery (REG 2). Lactate (B_La_) and rate of perceived exertion (RPE) were assessed immediately after exercise cessation, average resting heart rate (HR_rest_) was assessed as 5-min average during resting state. Average running HR (HR_run_) is provided as mean during each experimental condition. Graph metrics are revealed from 5-min resting EEG recordings and are presented as normalized values for the three frequency bands of theta (5–8 Hz), alpha-1 (8–10.5 Hz) and alpha-2 (10.5–13 Hz)

### EEG power analysis

#### Theta band

Significant main effects for CONDITION in the theta band were observed for parietal (*F* = 2.9, *p* = 0.020, partial eta^2^ = 0.160) and occipital ROI (*F* = 2.6, *p* = 0.031, partial eta^2^ = 0.149). Post hoc Bonferroni–Holm comparisons revealed no significant difference between any pairs of conditions.

#### Alpha-1 band

Significant main effects for CONDITION were observed for central (*F* = 2.5, *p* = 0.037, partial eta^2^ = 0.144), parietal (*F* = 4.4, *p* = 0.002, partial eta^2^ = 0.226) and occipital ROIs (*F* = 4.4, *p* = 0.009, partial eta^2^ = 0.227). For the parietal ROI, post hoc analysis revealed significantly increased alpha-1 power at ACUTE 2 (*p* = 0.03) and ACUTE 3 (*p* = 0.03) compared to PRE. For the occipital ROI, alpha-1 power at ACUTE 2 (*p* = 0.028) and ACUTE 3 (*p* = 0.015) were significantly higher compared to PRE.

#### Alpha-2 band

**Fig. 2:**
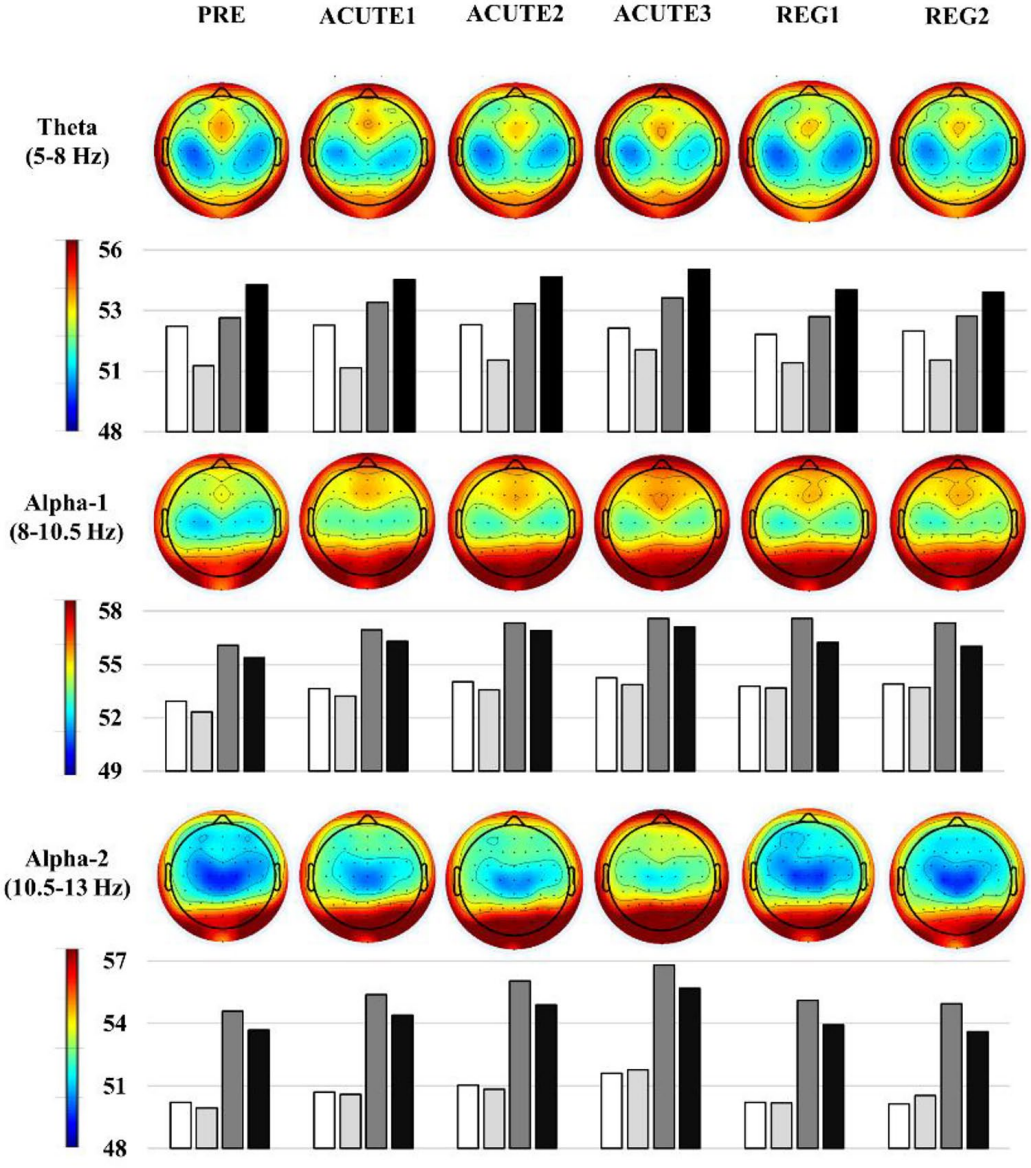
Overview of changes in topographical power distribution throughout the different experimental conditions. The experimental conditions refer to 5 min resting-state EEG assessment at rest (PRE), as well as directly following 10-min low-intensity running (ACUTE 1), 10-min moderate-intensity running (ACUTE 2), all-out exhaustive running (ACUTE 3), 8-min cool-down running (REG 1) and 10-min active recovery (REG 2). Global field values are presented as absolute power values log*-10 μV. Red colors indicate higher values while blue colors indicate lower total power values. Absolute mean values in regions of interest (ROI) are additionally represented as bars. *White bars* indicate frontal ROI, *light grey bars* indicate central ROI, dark grey bars indicate parietal ROI and *black bars* indicate occipital ROI

Significant main effects for CONDITION were observed for frontal (*F* = 6.1, *p* < 0.001, partial eta^2^ = 0.289), central (*F* = 6.3, *p* < 0.001, partial eta^2^ = 0.296), parietal (*F* = 8.5, *p* < 0.001, partial eta^2^ = 0.362) and occipital ROIs (*F* = 5.8, *p* = 0.004, partial eta^2^ = 0.280). Post hoc analysis revealed significantly increased alpha-2 power in the frontal ROI at ACUTE 3 compared to PRE (*p* = 0.039), REG 1 (*p* = 0.006) and REG 2 (*p* = 0.029). For the central ROI, alpha-2 power was significantly higher at ACUTE 3 compared to PRE (*p* = 0.011), REG 1 (*p* = 0.008) and REG 2 (*p* = 0.01) and at ACUTE 2 compared to REG 1(*p* = 0.008) and REG 2 (*p* = 0.01). For the parietal ROI, alpha-2 was significantly higher at ACUTE 3 compared to PRE (*p* = 0.005), ACUTE 1 (*p* = 0.012), REG 1 (p =  < 0.001) and REG 2 (*p* = 0.011) and for ACUTE 2 compared to PRE (*p* = 0.045), REG 1(*p* < 0.001) and REG 2 (*p* = 0.012). For the occipital ROI, alpha-2 was higher at ACUTE 3 compared to PRE (*p* < 0.001) and ACUTE 1 (*p* < 0.001). A visualization of topographical power changes and mean values throughout the six conditions is presented in Fig. 2.

### EEG network analysis

#### Theta band

Analysis of brain networks in the theta frequency band revealed a significant main effect for CONDITION on CC (*F* = 3.4, *p* = 0.008, partial eta^2^ = 0.185) and SWI (*F* = 2.8, *p* = 0.022, partial eta^2^ = 0.158). Post hoc Bonferroni–Holm corrected analysis revealed that CC was reduced at ACUTE 3 compared to REG 1 (*p* = 0.015). For SWI, post hoc Bonferroni–Holm analysis revealed significantly lower values for ACUTE 3 compared to REG 1 (*p* = 0.03).

#### Alpha-1 band

Brain network analysis revealed significant main effects for CONDITION on CC (*F* = 3.2, *p* = 0.036, partial eta^2^ = 0.178), PL (*F* = 2.7, *p* = 0.028, partial eta^2^ = 0.151) and SWI (*F* = 3.2, *p* = 0.042, partial eta^2^ = 0.174). Post hoc Bonferroni–Holm analysis corrected for multiple comparisons revealed no significant differences between any specific conditions.

#### Alpha-2 band

**Fig. 3:**
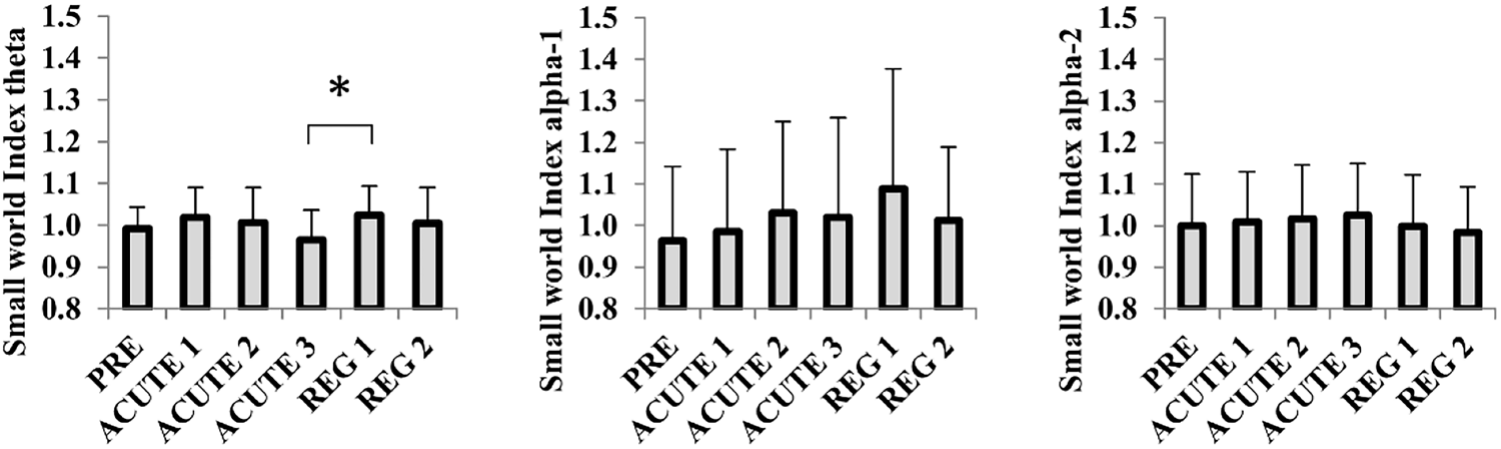
EEG-derived normalized Small-World-Index in theta, alpha-1 and alpha-2 frequency bands in response to an incremental exercise protocol on the treadmill. The post-exercise measurements refer to 5 min resting-state recordings following 10-min low-intensity running (ACUTE 1), 10-min moderate-intensity running (ACUTE 2), all-out exhaustive running (ACUTE 3), 8-min cool-down running (ACUTE 3) and 10-min active recovery (REG 2). Bars indicate mean graph outcomes per condition including standard deviation. Graph metrics are derived from 50 consecutive 8 s EEG resting-state epochs with 4 s overlap in sitting position with eyes open. * = significant post-hoc difference according to Bonferroni–Holm. Level of significance is set at *p* < 0.05

Brain network analysis revealed no main effect for CONDITION in the alpha-2 frequency band. An overview of graph measure changes throughout the six conditions is given in Fig. 3.

## Discussion

The purpose of this study was to explore intensity-dependent modulations in RSN efficiency in response to treadmill exercise. Therefore, we applied graph theory on intermittently assessed EEG resting-state data during an incremental treadmill protocol until voluntary exhaustion. The main findings demonstrate that exercise intensity modulates RSN efficiency in the theta band. By approaching voluntary exhaustion, a reduction in RSN efficiency was observed by means of network randomization, majorly induced by reduction in the clustering coefficient. In contrast, analysis of regional brain activity did not reveal intensity-dependent modulation, but a general increase in power values after exercise.

### Power analysis

Our analyses revealed significant increments in the alpha-1 and alpha-2 frequency band following exercise compared to rest, indicating the biggest impact of exercise on alpha-2 power in parietal brain regions. However, statistical tests failed to show significant differences between moderate-intensity and exhaustive exercise. In this regard, our findings are in accordance with previously published data reporting global changes in EEG power spectral density across different frequency bands after exercise. Additionally, despite the modulation of parietal alpha-2 power, no brain region revealed power changes with regard to exercise intensity, which is also in common with previous meta-analyses (Crabbe and Dishman 2004). These studies associated increased alpha power with increased task-related cortical inhibition, possibly indicating a state of task-induced brain in-activation during exercise. Behavioral observations propose that exercise and brain function interact in a dose–response relationship, assigning the most beneficial effect on brain function to moderate- to high-intensity exercise (Herold et al. 2019). In line with that, exhaustive exercise is associated with loss of distinct brain function, for instance, expressed by altered kinematics in cyclic motor tasks (Bassan et al. 2015; Bucher et al. 2018; Zory et al. 2009). Consequently, the analysis of modulations of regional brain activity does not reflect this intensity-dependent pattern. As neuroimaging findings suggest that the brain is organized as a large-scale network (Fox et al. 2005), not only the degree of activity of single neuronal patches, but rather their interconnectedness might be from interest. Therefore, we further explored RSN organization applying graph theory on the present EEG data.

### Theta network

The novel approach of our study was to describe RSN modulations in response to incremental exercise applying a graph theoretical approach. We observed significant modulations in the theta network, as SWI and in particular CC decreased in response to exhausting exercise. The SWI in the theta network was lower following voluntary exhaustion compared to all other post-running RSN assessments, even if statistical significance was only observed in comparison to the first recovery stage. The lack of further statistical differences between conditions may be caused by inter-individual differences in subjective and objective exercise responses at submaximal intensity. The state of voluntary exhaustion was underlined by perceptual and physiological assessments indicating RPE and HR values close to the individual maximum. Thus, a relationship between the physiological state of exhaustion and theta network randomization might be assumed. When perceptual and physiological load recovered in the consecutive stages of the experiment, theta network organization recovered as well.

In clinical investigations, a decrease in small-world characteristics in the theta network was associated with deficits in network efficiency in patients with cognitive deficits like Alzheimer’s disease and schizophrenia (Sun et al. 2019; Vecchio et al. 2017). Hereof, it is assumed that more long and less short connections reduce a networks efficiency, as information needs more steps to travel from one point of the network to any other point (Vecchio et al. 2017). Even if the absolute changes in brain network metrics are very small, functional connectomes rely on functional and structural connections (Shen et al. 2015). Therefore, intra-individual modulations brain graphs should not be expected to be drastic within a given session as structural connections do not change extensively within short time periods (Tozzi et al. 2020). Traditionally, theta oscillations are suggested to originate from the prefrontal cortex and reflect attentional involvement and executive function (Sauseng et al. 2005). A randomization of the theta network may therefore sub-serve reductions in attentional processing and executive functions, as observed for motor coordination tasks following exhaustive exercise (McMorris et al. 2015). Thus, exhaustion-induced randomization of the theta network may represent a short-term disturbance of executive brain function.

Even if previous studies majorly focused on the beneficial effects of exericse on brain function (Basso and Suzuki 2017; Kujach et al. 2018), some findings from exhausting exercise are in line with our findings of a randomization of the theta RSN and indicate a short time disturbance of brain function. For instance, brain imaging findings on exercise-induced reductions in frontal theta were associated with a simultaneous loss of knee movement control (Baumeister et al. 2012). Further, Porter et al. (2019) reported reduced frontal clustering in the theta band during a highly challenging cognitive-physical dual task paradigm at exhaustive exercise intensities, associated with a loss of cognitive performance. Behavioral observations suggest that concurring resources, resulting from co-existing metabolic and central nervous demands might be responsible for the phenomena of reduced brain function after exhaustive exercise loads (McMorris et al. 2015). Our findings of randomization of the theta network at rest after exhaustive exercise may support this suggestion, even if we were not able to control for behavioral consquences as we measured RSNs.

Furthermore, our data reveal that theta SWI does not change in response to moderate, but exhaustive exercise, suggesting an intensity-specific relationship between exercise and brain network efficiency. Such a modulatory effect of exercise intensity on brain network organization was already suggested by Schmitt et al. (2019), demonstrating up-regulations of attention-related brain networks in response to low-, but not high-intensity exercise. In this regard, the prefrontal cortex function has been shown to be mediated by dopamine and noradrenaline concentration in an inverted U-shaped pattern, indicating beneficial neuro-regulating effects at low to moderate, but detrimental effects at maximum stress conditions (Arnsten 2009). Consequently, a down-regulation of cortical excitability in the prefrontal cortex, mediated by acute increase in stress hormones after exhaustive exercise, may contribute to a loss of network efficiency and should be subject of future studies.

### Alpha-1 network

Next to the changes in the theta network, analysis of variance demonstrated a significant main effect in the alpha-1 network in response to exercise. Our data reveal an increase in network efficiency after ACUTE 2 and REG 1, but did not reveal significant post hoc differences between any of the conditions after correction for multiple comparisons. At ACUTE 2 and REG 1, average B_La_ values of ~ 3 mmol/l that lactate is accumulating but that most athletes were still in an aerobic steady-state situation. Inter-individually, lactate values ranged from 1 to 5 mmol/l and describe inter-individual physiological state below, close to or slightly above the so-called lactate threshold. It might be suggested that these inter-individual differences in physiological responses to submaximal exercise possibly contributed to the lack of significant systematic modulations in exercise-induced brain network changes.

Originally, alpha 1 oscillations are associated with thalamo-cortical traffic and are treated as markers of the excitability of cortical neurons (Klimesch 1999). Consequently, modulations in alpha 1 SWI might be associated with changes in efficiency in thalamo-cortical networks. The role of lactate accumulation on brain function has generally been researched in behavioral investigations and demonstrates that increased blood lactate is related to improvements in brain functions (Kujach et al. 2018; Takehara et al. 2017). On a neurochemical base, Magistretti and Allaman (2018) explained that lactate can inter alia increase the excitability of cortical neurons and function as a signaling molecule in the central nervous system. However, fMRI findings demonstrate increased interconnectedness in sensorimotor networks following low- to moderate-intensity exercise, while exhaustive exercise led to loss of interconnectedness (Rajab et al. 2014; Schmitt et al. 2019). Furthermore, Robertson and Marino (2015) reported that brain activity in the frontal cortex decreases when passing individual aerobic thresholds. In line with that, a few studies found associations between increases in blood lactate concentration and reductions in brain function (Coco et al. 2016; Perciavalle et al. 2015). Therefore, it might be suggested that submaximal exercise and its induced metabolic changes may modulate brain network organization by changing neural excitability bidirectional depending on individual aerobic capacity and internal load.

### Alpha-2 network

In the alpha-2 network, no statistically significant changes in response to incremental treadmill running were observed. Alpha-2 frequencies are typically associated with task-related processing and are suggested to reveal from cortico–cortical interactions induced by cognitive and/ or sensorimotor demands (Klimesch 1999). Therefore, it might be speculated that these frequencies are not modulated by exercise during rest.

### Brain mechanisms underlying modulations of brain networks

The present findings on exercise-induced brain network changes suggest that graph theory might be a powerful tool to display modulations of inherent cortical processes. Based on our explorative findings, we observed an intensity-dependent pattern of brain network modulation, indicating a loss of efficiency in attention-related networks induced by exhaustion. Recently published findings from fMRI (Schmitt et al. 2019) and EEG (Tamburro et al. 2020) reporting intensity-dependent modulations of brain networks support our findings. We suggest that these modulatory patterns possibly contribute to intensity-dependent alterations of human behavior after acute exercise (Herold et al. 2019). Hereof, a loss of RSN efficiency in the theta network after exhaustive exercise may possibly explain reductions observed during both motor tasks as well as complex cognitive tasks (McMorris et al. 2015; Zimmer et al. 2017) by reducing the brain network’s capability to exchange information.

Taken together, monitoring RSN efficiency by means of graph theory might help to better understand exercise-induced modulations of human behavior. With regard to sport and exercise, the observed loss of RSN efficiency may serve to explain performance decrements during or after exhaustive exercise bouts (Bassan et al. 2015; Bucher et al. 2018; Zory et al. 2009). Graph measures obtained from athletes during rest may therefore reveal complementary information on an athlete’s readiness to perform motor coordination tasks, displaying the brains responsiveness to external stimuli (Raichle 2011). Especially in sport and exercise where a high degree of attentional focus and information processing is required under intense cardiovascular load, e.g. biathlon (Luchsinger et al. 2016), more elaborated information on brain network efficiency could help to control and individualize training protocols and pacing strategies. Thus, next to subjective information on athlete’s readiness and recovery (Heidari et al. 2019), EEG may provide an objective alternative to these measures. Future studies on brain networks in exercise settings need to further elaborate contextual variables of exercise like training volume, training modality and athletes’ fitness level. Furthermore, upcoming studies incorporating graph measures may extend current perspectives on the concept of neural efficiency in athletes (Del Percio et al. 2009; Ludyga et al. 2016) which to date solely analyze electro-cortical oscillations from an activity, but not from a connectivity point of view.

### Limitations

Even if our findings are promising and display time courses that are in line with behavioral observations investigating the effect of exercise on brain functions, some limitations must be stated. The primary limitation is the lack of behavioral correlates for the observed network changes. Therefore, the functional consequences of a loss of brain network efficiency are based on plausibility derived from findings of previously published studies. However, RSN organization is associated with brain function (Shaw et al. 2015) and seem to be a valuable aspect in detecting the modulating effect of exhaustive exercise on motor coordination. Future studies investigating modulations in RSN efficiency should focus on an external validation of brain outcomes. Furthermore, data must be interpreted with caution and should be carefully generalized due to the study’s explorative character in combination with a relatively small sample size (*n* = 16). Furthermore, brain graphs need to be treated as mathematical representations of functional brain networks (Stam et al. 2014). Global CC and PL are general measures describing network characteristics that do not allow assumptions on specific architectural changes within the brain network. For this purpose, a comparison of the present findings with fMRI findings on regional changes in RSN should be restricted on broad functional modulations of brain networks. Even if the wPLI is less sensitive to volume conduction (Vinck et al. 2011), the possible influence of the EEG inherent inverted-source problem on brain graph reconstruction needs to be considered, as EEG signals represent a mixture of multiple electrical sources. In line with different FC estimation methods, preserving zero-lag correlations other than the wPLI may result in alternating resting-state network configuration compared to the present findings (Rizkallah et al. 2020). Next to the comparison with regard to the different neuroimaging techniques applied, Schmitt et al. (2019) already mentioned that the comparability of different studies is hampered by the lack of standardization in exercise protocols. Therefore, direct comparisons should be avoided as it remains unclear how different exercise modalities, e.g. cycling and running, or different exercise intensities and volumes affect RSNs. In addition to that, the impact of fitness level on the modulatory effect of exercise on brain function needs to be considered. Even if we investigated a group of physically active students, we observed a heterogeneous level of fitness as derived from VO_2 peak_ assessment and inter-individual differences in physiological responses to submaximal exercise. In this regard, Ludyga et al. (2016) reported that modulations of cortical activity during cycling were modulated by the fitness level of the participants, suggesting lower responses for trained individuals in line with the neural efficiency hypothesis (Del Percio et al. 2009; Ludyga et al. 2016). Therefore, future studies on brain network efficiency need to investigate the protective effect of fitness levels on RSN efficiency.

## Conclusion

The present study demonstrates that RSN efficiency could be modulated by acute exercise as exhausting exercise seems to reduce network efficiency. The assessment of RSNs due to EEG may therefore provide valuable information on brain function, representing brain characteristics related to information transfer and cortical interconnectedness. Taking into account that exercise load seems to affect motor coordination, reductions of RSN efficiency may contribute to this phenomena impairing the capability of the central nervous system to successfully transfer and process task-relevant information.

By assessing the RSN, future studies may address both acute and chronic effects of exercise on brain function in exercise-related settings. Hereof, more advanced statistical prediction models may help to identify relationships between resting-state brain network configuration and sport performance. To gain a better understanding on how exercise impacts brain function, future studies need to address contextual variables like exercise modality, exercise volume and exercise intensity as well as internal variables, such as age, fitness level or disease. Further findings would not only help coaches to manage training loads with regards to athletes’ readiness, but also recreational athletes or clinicians to optimize the use of exercise as a modulator of inherent brain function.

## Abbreviations

EEG: Electroencephalography
RSN: Resting-state network
CC: Clustering coefficient
PL: Characteristic path length
SWI: Small world index
VO_2 peak_: Peak oxygen consumption
HR: Heart rate
BS: Borg scale
B_La_: Blood lactate concentration
ANOVA: Analysis of variances
wPLI: Weighted phase-lag index
